# Protein aggregation triggers a declining libido in elder yeasts that still have a lust for life

**DOI:** 10.15698/mic2017.06.578

**Published:** 2017-05-28

**Authors:** Fabrice Caudron

**Affiliations:** 1 Randall Division of Cell and Molecular Biophysics, King’s College London.

**Keywords:** mnemon, prion, ageing, old cells sterility, asymmetric cell division, protein aggregation, budding yeast

## Abstract

Many organisms have to face a physiological decline that is associated with age. Humans and even budding yeast accumulate scars and cellular damages. A single yeast cell can only produce a limited number of daughter cells and thus has a finite replicative lifespan. Many studies have now identified molecular ageing factors and defects in organelle functions linked to the ageing process. However, at the cellular level, the most striking phenotype of yeast elders is their loss of mating ability. This sterility in old cells has been linked to a loss of response to mating pheromone, the peptide that haploid yeast cells send to opposite mating type cells in order to signal their presence and readiness to mate. Our results (Schlissel *et al*., 2017) demonstrate that old cells are unable to respond to mating pheromone due to age-induced aggregation of the protein Whi3. We recently discovered that Whi3 changes conformation and coalesces when cells experience and memorise a deceptive mating attempt. Together, these results prompt the question of how proteins physiologically aggregating behave during ageing, induce age associated phenotypes and influence the ageing process itself.

## THE STERILITY OF OLD YEAST MOTHER CELLS 

The most striking phenotype that is associated with advanced ageing in budding yeast is the loss of old cells’ mating ability. When a single cell is challenged with a mating partner, it stops its cell cycle in the G1 phase and grows - ‘shmoos’ - towards the mating partner. This arrest is mediated by a diffusible mating pheromone that triggers a signalling cascade and results in a reprogramming of cell fate. Pheromone induced arrest is very efficient and all young cells will form shmoos and successfully mate to form diploid cells. This scenario is best illustrated when the four spores of a tetrad mate pairwise upon germination. However, mating fails when an old cell is placed next to a young cell. This mating failure was thought to reflect a defect in heterochromatin formation and gene silencing. Indeed, desilencing of the Hidden Mating type Loci results in cells behaving as pseudo-diploids cells that do not secrete pheromone nor express the pheromone receptor. Consequently, these cells do not shmoo and mate. Using diverse modern sensitive assays, we found that silencing was not impaired in old cells of the mostly used laboratory strain genetic backgrounds. Using increasing concentrations of pheromone, we realised that old cells were not completely irresponsive to pheromone, but they required more than young cells to initiate a shmoo. Interestingly, most of the daughter cells of old unresponsive cells did shmoo immediately after birth. This asymmetric mode of inheritance and the requirement for a higher pheromone concentration to respond prompted us to consider the involvement of Whi3 in the old cells sterility phenotype.

## AGGREGATION OF THE Whi3 MNEMON 

**Figure 1:**
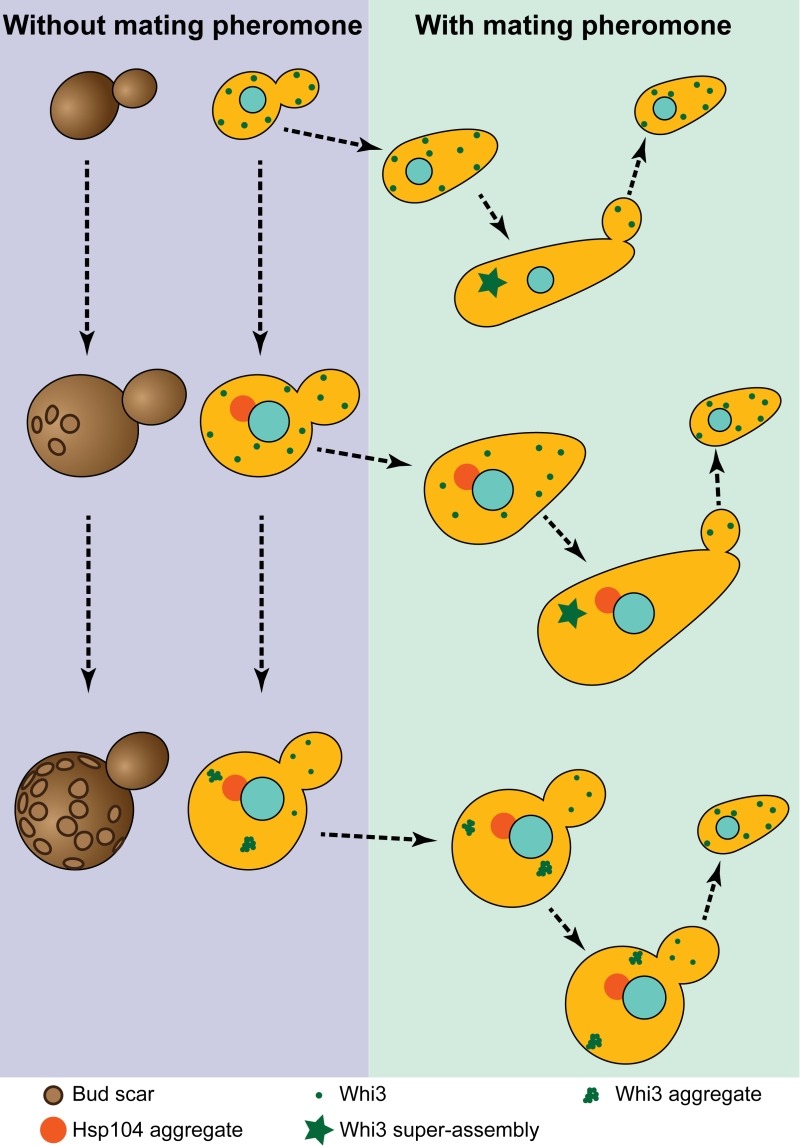
FIGURE 1: The different ages of budding yeast. Young cells (no bud scars - left, small Whi3 puncta - green dots) shmoo in response to pheromone (right). In the absence of a mating partner, young cells escape the pheromone arrest due to Whi3 super-assembly (green star). Their daughters are born naïve and respond to pheromone. Middle age cells behave similarly to young cells but already accumulate an age induced protein deposit enriched in Hsp104 (red dot). Very old cells (many bud scars, left) still have an Hsp104 aggregate, but also display aggregated Whi3. When exposed to low pheromone concentration very old cells keep on dividing while their daughters which do not inherit Whi3 aggregates are still born naïve and shmoo.

Whi3 is a mRNA binding protein that inhibits translation of many mRNAs, including the G1 cyclin Cln3 mRNA. Through its inhibition of Cln3, Whi3 contributes to controlling the decision to enter the cell cycle or shmoo in response to pheromone. Whi3 sequence also features two prion-like domains that confer to Whi3 the ability to change conformation. Remarkably, this conformational change is accompanied by a functional change and Cln3 mRNA translation inhibition is released. Thus, when Whi3 changes conformation, cells can enter the cell cycle and produce daughter cells even when pheromone signalling is active (Figure 1). We observed that this conformational change correlates with Whi3 coalescence to super-assemblies. Since Whi3 super-assemblies are inherited asymmetrically, daughter cells are born naïve and shmoo in response to pheromone.

Because Whi3 shares many similarities with prions (prion-like domains, conformational change associated with a functional change, super-assembly), but has the different properties of being asymmetrically inherited during cell division and of being induced by a specific signal (pheromone), we termed Whi3 the founding member of mnemons. Recently, we discovered that Whi3 aggregates in old cell. Moreover, old cells expressing an allele of Whi3 that lacks one of its prion-like domains were still able to shmoo in response to pheromone. These results support a scenario in which Whi3 aggregates during ageing, releasing its inhibition on Cln3 mRNA and thus impairing the ability of old cells to respond to pheromone.

## PROTEIN AGGREGATION DURING AGEING

Whi3 is not the only protein to aggregate during ageing. Very early on, the disaggregase Hsp104 accumulates in the form of a single focus that is also enriched in other protein chaperones such as the Hsp70 family member Ssa1 or the small heat shock protein Hsp42. However, what other proteins beyond chaperones accumulate at these age-induced protein deposits is unknown. The protein Sup35 that form the prion [PSI+] does accumulate there, but for example the other protein Rnq1 forming the [RNQ+] prion does not. We observed Whi3 aggregating in rather old cells, probably those that are close to the dawn of their life. Thus, it is unlikely that a large proportion of Whi3 accumulates in early appearing age-induced protein deposits. These results clearly call for a need to better understand the determinants of age-induced protein aggregation. The prion form of Sup35 is induced stochastically while Whi3 conformational change is triggered by pheromone response. One hypothesis is that conformational changes of mnemons could be more controlled than those of classical prions. Potentially, a tighter inhibition on mnemon fold changes may also play an important role in their asymmetric mode of inheritance.

Another interesting aspect may lie in the concentration of the proteins of interest. Sup35 is at least 10 fold more abundant than Whi3 that does not aggregate early on during ageing. Rnq1 is also less abundant, but whether it aggregates in very old cells is unknown. Thus, an hypothesis may be that low-abundant proteins aggregate later in ageing than high abundant ones.

## THE COST OF CONFORMATIONAL FLEXIBILITY?

The focus of many studies on protein aggregation has mainly been driven by the use of model proteins, which were either mutant proteins or overexpressed exogenous proteins or truncations of endogenous proteins. With the development of genetic systems such as the mother enrichment program or microfluidic devices, we have now the possibility to explore the aggregation of proteins expressed at their endogenous levels in a systematic manner. Some proteins are under more intense scrutiny because they harbour prion-like, glutamine- and asparagine-rich, domains similar to those found in proteins aggregating in devastating neurodegenerative diseases such as Parkinson’s or Alzheimer’s diseases. However, recent work in yeast proposed that self-templating conformational domains may be more diverse than anticipated. Many proteins containing a domain with an intrinsically disordered nature can have long-lasting prion-like properties when transiently over-expressed. Therefore, the wealth of proteins being able to change conformation and thus being at risk of aggregating during ageing is probably more diverse than that of classical prion-like domain containing proteins. It will be decisive to understand whether proteins that make use of conformational change to sustain long-term adaptations and cellular memories do pay the cost of their fold flexibility by aggregating during ageing. Many of these proteins lie at important hubs of cellular pathways and are for example often linked to mRNA biology. Whi3 is thought to regulate the levels of hundreds of mRNAs either directly or indirectly, thus its aggregation and that of other mRNA binding proteins may broadly influence the behaviour of a cell during ageing. Remarkably, cells expressing an allele of *WHI3* lacking one of its prion-like domains (*whi3*-∆*pQ*) were slightly longer-lived than wild-type.

## EXPLORING DIVERSITY WITH ELDERS?

Altogether, aggregation of proteins seems to occur throughout the life span of budding yeast cells, starting soon after they produce their first daughter cell. Yet, at the end of their lifespan, more diverse proteins will aggregate than at the beginning. Since aggregation may be an intrinsically stochastic process at the proteome level, its effect on mRNAs expression for example will be highly variable. Thus, old cells may represent a reservoir of cells within a younger population with very diverse expression profiles that could be assimilated to an extended version of noise in gene expression. Therefore, protein aggregation, which was recently shown to be protective for the aggregating proteins themselves in young yeast cells exposed to thermal stress, may also be a diversification strategy established in old cells to cope with a fluctuating environment. Since in all these cases, protein aggregates are kept in the mother cell during cell division, this diversity would only be discrete in the whole population. Modern single cell microscopy and analysis tools will probably reveal some unexpected aspects of cellular ageing in the future.

